# A C-terminal Pfs48/45 malaria transmission-blocking vaccine candidate produced in the baculovirus expression system

**DOI:** 10.1038/s41598-019-57384-w

**Published:** 2020-01-15

**Authors:** Shwu-Maan Lee, John M. Hickey, Kazutoyo Miura, Sangeeta B. Joshi, David B. Volkin, C. Richter King, Jordan L. Plieskatt

**Affiliations:** 1 PATH’s Malaria Vaccine Initiative (MVI), 455 Massachusetts Avenue NW, Suite 1000, Washington, DC 20001-2621 USA; 2 Department of Pharmaceutical Chemistry, Vaccine Analytics and Formulation Center, University of Kansas, Lawrence, KS 66047 USA; 3 Laboratory of Malaria and Vector Research, National Institute of Allergy and Infectious Diseases, National Institutes of Health, Rockville, MD 20852 USA

**Keywords:** Liquid chromatography, Recombinant vaccine, Expression systems, Glycosylation, Malaria

## Abstract

The *Plasmodium falciparum* gametocyte surface protein, Pfs48/45, is a potential target for malaria transmission-blocking vaccines. However, due to its size and complexity, expression of the full-length protein has been difficult, leading to focus on the C-terminal six cysteine domain (6C) with the use of fusion proteins to facilitate expression and folding. In this study, we utilized the baculovirus system to evaluate the expression of three Pfs48/45 proteins including the full-length protein, the 6C domain fragment and the 6C domain mutant to prevent glycosylation. Expression of the recombinant Pfs48/45 proteins was conducted in super Sf9 cells combined with the use of tunicamycin to prevent *N*-glycosylation. The proteins were then evaluated as immunogens in mice to demonstrate the induction of functionally active polyclonal antibody responses as measured in the standard membrane feeding assay (SMFA). Only the 6C protein was found to exhibit significant transmission-reducing activity. Further characterization of the biologically active 6C protein demonstrated it was homogeneous in terms of size, charge, conformation, absence of glycosylation, and containing proper disulfide bond pairings. This study presents an alternative expression system, without the need of a fusion protein partner, for the Pfs48/45 6C protein fragment including further evaluation as a potential transmission-blocking vaccine candidate.

## Introduction

Malaria transmission-blocking vaccines (TBVs) are being evaluated for their potential to serve as a supplemental tool to accelerate the elimination of malaria. TBVs function via the induction of antibodies that block the fertilization or the penetration of the mosquito midgut by sexual stage parasite, thereby breaking the cycle of parasite transmission between human and mosquito hosts^1–3^. Several sexual stage antigens (e.g., Pfs25, Pfs230 and Pfs48/45) have been identified as promising TBV targets for research or early stage clinical development. In clinical testing, Pfs25, when delivered as nanoparticles^4^, induced functional antibodies. However, the responses were of low magnitude and short-lived^5,6^. In recent years, development attention has shifted to the TBV candidates Pfs230 and Pfs48/45 in view of the poor outcomes associated with Pfs25-based constructs and the fact that sera from *Plasmodium* infected individuals are associated with transmission-blocking antibodies attributable to these gametocyte surface antigens^7^. Moreover, affinity purified anti-Pfs48/45 and anti-Pfs230 antibodies from naturally exposed individuals can prevent the transmission of cultured *P. falciparum* gametocytes^8^, when concentrated nine times the physiological concentration, thereby demonstrating the functionality of these natural antibody responses and the potential for these antigens in TBV development.

While the preclinical^9–11^ and clinical (NCT 02942277) development of Pfs230 is in progress, the development of Pfs48/45 as a TBV candidate has remained challenging. The Pfs48/45 protein, expressed on the surface of gametocyte, serves an essential role in the male gamete fertility^12^ and belongs to the same cysteine-rich structural family as Pfs230^13^. Consisting of 448 amino acids in its full-length form, the native Pfs48/45 sequence also contains a signal sequence, three cysteine motifs organized as one and half double domains, a putative glycosylphosphatidylinositol anchor and seven potential *N*-glycosylation sites^14^.

The expression of soluble and properly folded full-length Pfs48/45 has presented a challenge for recombinant expression systems, thus leading to focused efforts on expression of individual domains of Pfs48/45. This difficulty in expression has largely been attributed to its three-domain structure and large number of disulfide bonds (16 cysteines arranged as eight disulfide pairs). Conformational monoclonal antibodies are available and have facilitated the design and characterization of recombinant Pfs48/45 proteins. For example, rat monoclonal antibody 85RF45.1, developed by Roeffen *et al*. using the native gametocyte antigen, recognizes the C-terminal epitope I^15^ and possesses potent transmission-blocking activities (TBA) with an IC_80_ of 1–3 µg/mL as measured in the SMFA^16^. To design a recombinant TBV, Outchkourov *et al*. analyzed the transmission-blocking (TB) epitopes on the Pfs48/45 protein and determined the C-terminal six-cysteine module termed 6C was recognized by the 85RF45.1 monoclonal antibody^17^. An extended design including the middle four-cysteine module and the C-terminal 6C, designated as 10C, has also been attempted. When the 10C protein was fused with maltose binding protein and expressed in the *E.coli* periplasmic space^18^, the resulting protein was functionally active in mice, however, the overall yield (1 mg/L culture) was too low for vaccine development.

Numerous expression systems have since been explored to produce a Pfs48/45 protein, however, the reported yields and purity of properly folded protein have not been satisfactory for the resulting protein to be considered a vaccine candidate^19^. Bacterial expression systems have generally been preferred, given their ability to produce a non-glycosylated protein, but have also been challenging given the complex structural nature of Pfs48/45. Singh *et al*. reported successful expression and characterization of 6C as a chimera fused with N-terminal region of GLURP (R0) and a C-terminal his tag in the *Lactococcus lactis* system^20^. Biochemical characterization has also been well reported for R0.6C, with a final purified yield of 25 mg/L culture as well as the ability to elicit functional antibodies in rats^20^. While this approach is promising, we sought to generate Pfs48/45 antigens that might focus the immune response onto the 6C region alone without fusion partners.

Expression in eukaryotic systems has also been reported and attempted for Pfs48/45 based antigens. Such systems add additional complexity since *Plasmodium* parasites lack *N*-linked glycosylation machinery^21^ but Pfs48/45 contains seven potential *N*-glycosylation sites when expressed in eukaryotic systems. Thus, the protein could be aberrantly glycosylated impacting the ability to generate functional antibodies. Evidence suggested by Kapula *et al*. that expression in human embryonic kidney (HEK) 293 cells of unmodified Pfs48/45 (48/45 +Ngln) and modified Pfs48/45 that removes glycosylation sites (48/45-NGln) could be achieved^22^. Unfortunately, the resulting recombinant protein contained high molecular weight aggregates, with modified (-NGln) demonstrating no significant TBA. This work demonstrated the importance of the primary sequence integrity for this protein. Mamedov *et al*. co-expressed Pfs48/45 with Endoglycosidase H (Endo H) in the plant based expression system and the recombinant deglycosylated Pfs48/45 generated functional antibodies in mice^23^. This work demonstrated the feasibility of recombinant expression of full-length Pfs48/45 protein.

Recently, Kundu *et al*. expressed 6C in HEK cells for the study of its binding to 85RF45.1^16^ and Lennartz *et al*. expressed full-length Pfs48/45, 10C and 6C fragments in the Drosophila S2 expression system^24^. The full-length Pfs48/45 was used to raise a panel of mouse monoclonal antibodies and the fragments utilized to map the binding regions of those resulting antibodies^24^. These studies show that it is feasible to express 6C without a fusion partner.

In this study, we explore the use of an alternative expression system to generate recombinant Pfs48/45 proteins. Previously, we demonstrated the baculovirus system could be used to produce properly folded Pfs25 and an N-terminal fragment of Pfs230 at reasonable yields with high purity, and more importantly, with functional activity^9,10,25–27^. In view of this success, we tested whether full-length Pfs48/45, the 6C domain protein and the 6C mutant can be produced using this system, and whether the expressed proteins retained native conformation to elicit TBA. We report here the biochemical and immunological characterization of the expressed proteins, and the results suggest that the 6C fragment may be feasible from a vaccine development perspective given further optimization. Moreover, the well characterized and homogeneous 6C fragment may also have utility as a reference reagent to support malaria vaccine development.

## Results

### Baculovirus produces full-length Pfs48/45 and 6C fragments

The design of baculovirus expression constructs was based on previous work on the Pfs230C1 recombinant protein^9^. A signal peptide (MKFLVNVALVFMVVYISYIYAD) to facilitate secretion through ER pathway, and a six histidine C-terminal tag were included to facilitate protein expression and purification of the full-length Pfs48/45 (aa 28-427), 6C fragment (aa 291–427) and 6C Mutant (aa 291–427 with N299Q and N303D mutations). In this report, these three recombinant proteins are designated Pfs48/45-FL, 6C and 6C-Mut, respectively, as derived from baculovirus expression. The insect cells used in the baculovirus system have active glycosylation machinery while the evidence to date is that *P. falciparum* parasites do not^21^. To address the glycosylation propensity of the Pfs48/45 molecule during insect cell production, two approaches were undertaken. In an effort to minimize modifications (e.g., mutations) to the native gene sequence, the primary approach was to include tunicamycin, an antibiotic, in the expression culture as it has been demonstrated to effectively inhibit *N*-glycosylation in the insect/baculovirus expression system^28,29^. The secondary approach to eliminate *N*-glycosylation in the 6C fragment was to mutate the two high probability *N*-glycosylation sites at positions N299 and N303 in the 6C construct (termed 6C-Mut).

**Figure 1:**
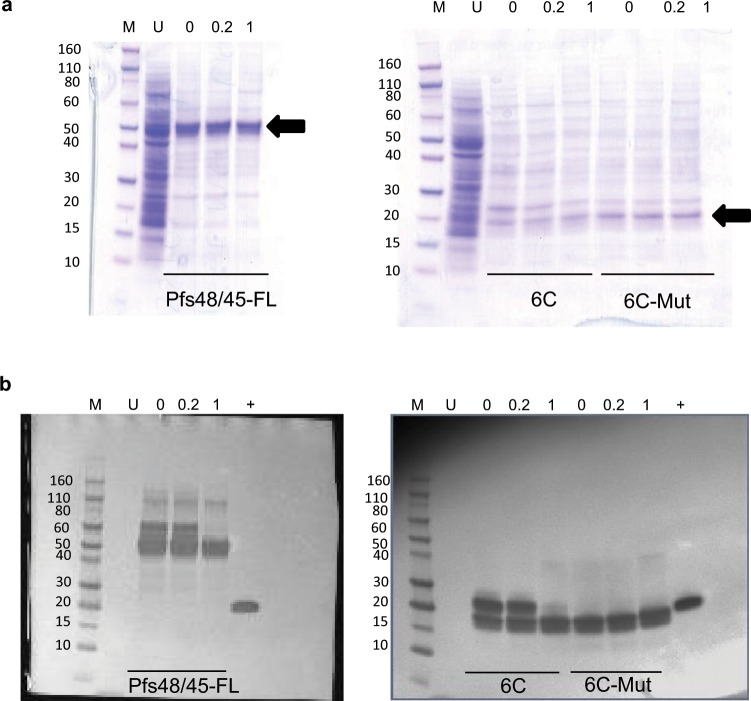
SDS-PAGE and Western blot analysis of Pfs48/45 baculovirus/insect cell pellets. Insect cell pellets from 96 hours expression with Pfs48/45-FL, 6C and 6C-Mut were evaluated by (**a)** Two separate Coomassie stained gels and **(b)** Two separate Western blots with anti-His antibody. M: Marker (kDa); U: Uninfected control at 96 hours (pellet); 0, 0.2, 1: Tunicamycin concentrations in µg/mL; +: Irrelevant his-tag protein positive control. Gels and Western blots were run at the same time and in similar manner. SDS-PAGE gels stained with Coomassie (**a**) were cropped to areas of interest (Left panel to first five lanes) at time of image acquisition. Images were not further manipulated.

To test the initial feasibility of expression in the presence of tunicamycin, 30 mL culture was infected with baculovirus at 1 MOI (multiplicity of infection) and tunicamycin was added to a final concentration of 0, 0.2 or 1 µg/mL. It should be noted, as shown in Supplementary Table S1, that tunicamycin significantly inhibited cell growth, and this inhibition may have impacted protein expression overall. The recombinant proteins were not expressed in the supernatant but were rather located in cell pellets as indicated by SDS-PAGE and anti-his Western (Fig. 1). All three proteins were expressed at the expected molecular weight (Fig. 1a). However, the Pfs48/45-FL and 6C protein both presented as double bands (Fig. 1b) in the presence of 0 or 0.2 µg/mL tunicamycin, likely the result of glycosylation. At tunicamycin concentrations of 1 µg/mL, the glycosylation appeared completely inhibited as the proteins presented as a single band (Fig. 1b). Tunicamycin did not impact the banding pattern of 6C-Mut (Fig. 1b), which presented as a single band under all conditions, as *N*-glycosylation was already likely inhibited through the mutation of the two high probability *N*-glycosylation sites.

To produce sufficient recombinant protein for further characterization and *in vivo* evaluation, the insect culture was scaled up to 10 L, and 1 µg/mL tunicamycin was selected for addition during infection, based on the results of small-scale experiments. The proteins were successfully extracted from homogenized cell pellets in the presence of 2% sarkosyl and purified by IMAC (immobilized metal affinity column) and size-exclusion chromatography into a formulation buffer of 20 mM HEPES, 150 mM NaCl, 0.2% Tween 80, pH 7.5. The initial process, as presented here yielded <3 mg of purified protein per liter of culture for 6C and 6C-Mut. These yields were considered low, but sufficient to conduct initial evaluations. The Pfs48/45-FL was produced in even smaller quantities (<0.1 mg/L culture). Due to the much higher yield of the 6C fragment, we now prefer this as a candidate antigen, however, production of the full-length was sufficient to be used as a comparator in subsequent *in vivo* studies. Efforts to improve expression were not explored further in the study reported here, and yield optimization would be required for further development as a TBV antigen. This however remains plausible given our past experience in process optimization^10^, and with new focus on maximizing yield from the cell pellet, knowing that a homogeneous properly disulfide-paired protein can result.

**Figure 2:**
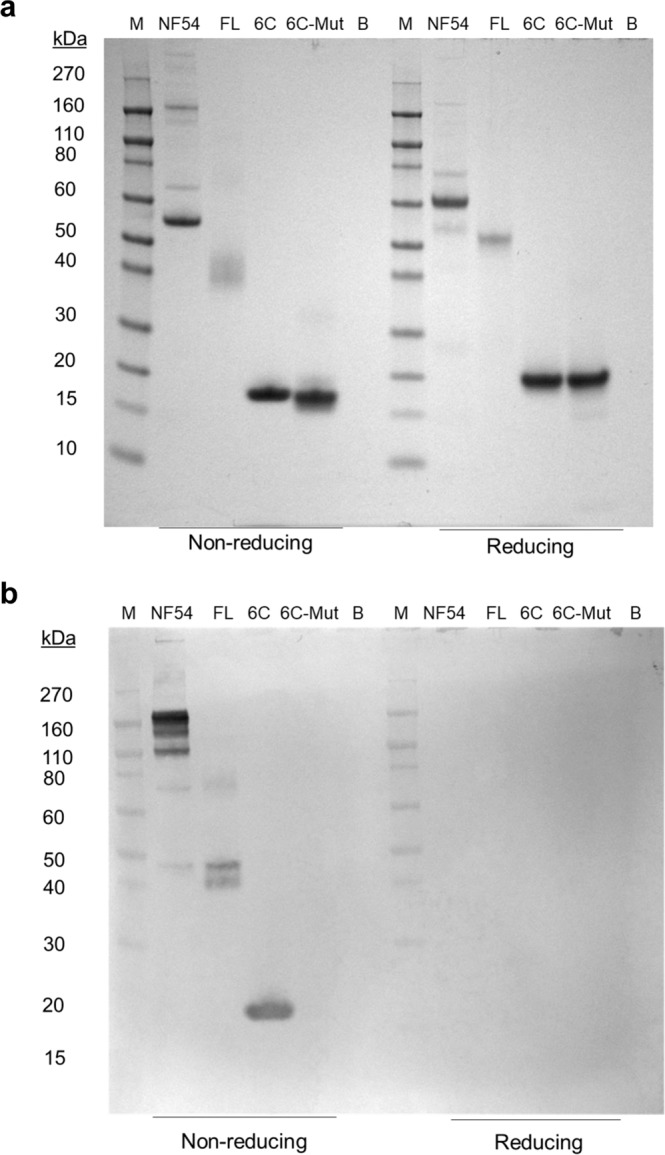
SDS-PAGE and Western blot analysis of purified Pfs48/45 recombinant proteins. Purified proteins were analyzed via SDS-PAGE followed by **(a)** Coomassie staining and **(b)** Western blot with a functional monoclonal antibody (85RF45.1). Lanes loaded under non-reducing and reducing conditions (M) Marker; (B) Blank; (NF54) NF54 parasite extract as positive control; (FL) Pfs48/45-FL; 6C and 6C-Mut proteins as labeled. Both (a: Coomassie stained and b: transfer for Western blot) were loaded in an identical manner and run at the same time. Images were cropped to areas of interest (Gel or membrane edges) at time of image acquisition. Images were not further manipulated.

The 6C and 6C-Mut proteins were >90% pure by SDS-PAGE and densitometry and Pfs48/45-FL was >80% pure (Fig. 2a). To determine whether the important epitope I was preserved, the proteins were also analyzed via Western blot with the 85RF45.1 monoclonal antibody (Fig. 2b). Native Pfs48/45, present in NF54 parasite extract as well as the recombinant Pfs48/45-FL and 6C proteins were successfully recognized by this reduction sensitive monoclonal, indicating this functional epitope was preserved. However, the 6C-Mut protein was not recognized (Fig. 2b) by 85RF45.1, likely indicating that the mutation of two *N*-glycosylation sites disrupted epitope I conformation.

### Baculovirus expressed 6C fragment elicits functional antibodies

**Figure 3:**
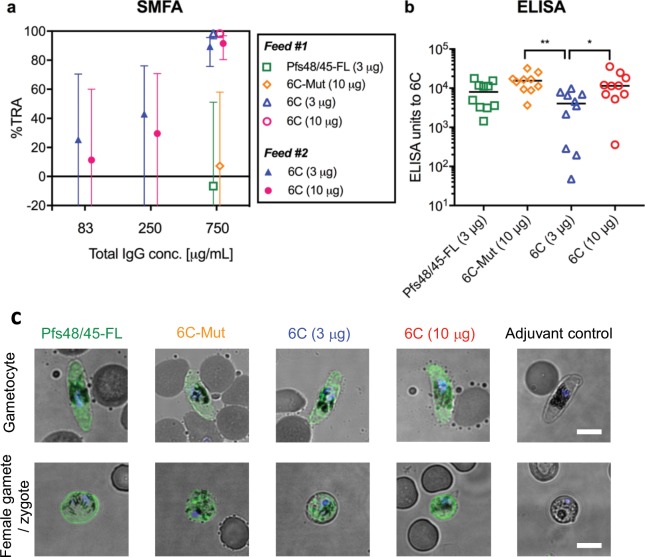
Immunological evaluations for recombinant Pfs48/45 constructs. Anti-Pfs48/45 antibodies were generated in CD-1 mice immunized with Pfs48/45-FL (3 µg), 6C-Mut (10 µg), or 6C (3 or 10 µg) recombinant protein. **(a)** To evaluate biological activity of the induced antibodies by SMFA, an equal volume pooled antiserum sample was made for each group, and Protein G purified IgG tested at 750 μg/mL in the initial assay (Feed #1). Anti-6C IgGs were retested in a second assay at 750, 250, and 83 μg/mL (Feed #2). The best estimate and the 95% CI of % inhibition (%TRA) for each test IgG at each test condition are shown. **(b)** Anti-6C ELISA units were determined for individual antiserum samples. Individual and median ELISA units are shown. A significant difference among the four groups (p=0.004 by a Kruskal-Wallis test) was determined, and following Dunn’s multiple comparison tests revealed that there were significant differences between 6C-Mut (10 µg) and 6C (3 µg) groups (p = 0.004), and between 6C (3 µg) and 6C (10 µg) groups (p = 0.037). (**c**) Immunofluorescence assay with fixed and permeabilized *P. falciparum* sexual-stage parasites. Purified total IgGs, which were used for SMFA, were incubated at 1 µg/mL with fixed mature gametocytes, gametes and zygotes. The antibody reactivity to the parasites are shown in green (Alexa Fluor 488), and DNA in blue (DAPI; 4′,6-diamidino-2-phenylindole). The scale bar represents 5 µm.

To test if the recombinant proteins represented the native conformation and therefore could elicit functional antibodies, a mouse immunogenicity study was conducted on all three baculovirus Pfs48/45 proteins formulated with the water-in-oil emulsion adjuvant Montanide ISA720. Antibody responses against each immunogen were determined by ELISA, and all recombinant proteins were immunogenic with median ELISA titer of >4,000 units and negative controls showing undetectable level of response (<18 ELISA units) against all three proteins. Biological activity of induced antibodies was then evaluated by SMFA using purified IgG derived from serum pool from all mice in each immunization group. In the initial assay (Fig. 3a and Supplementary Table S2, Feed #1), all purified IgGs (one pooled sample per group of 10 mice) were tested at 750 μg/mL. The IgGs from 6C (3 µg) and 6C (10 µg) groups were associated with significant reductions in oocyst density (>98% reduction, p <0.001 compared to adjuvant only negative control for both), while the IgGs from Pfs48/45-FL (3 µg) and 6C-Mut (10 µg) groups showed <10% reduction (p > 0.87). To confirm the functional activities of anti-6C IgGs, the two samples were further tested at three concentrations in an independent assay (Fig. 3a and Supplementary Table S2, Feed #2). Significant reduction in oocyst density was reproduced by IgGs tested at 750 μg/mL (>89% reduction, p < 0.001). Although percentage reductions at lower concentrations (250 and 83 µg/mL) did not reach significant level for both IgGs, a trend toward dose-dependent oocyst density reductions was observed.

The antisera raised against Pfs48/45-FL and 6C-Mut was then tested for reactivity via ELISA (Fig. 3b) to the 6C protein alone, the only immunogen which elicited biologically active antibodies. The anti-Pfs48/45-FL and anti-6C-Mut antisera both reacted to the 6C protein. To further investigate the mechanism of negative SMFA for anti-Pfs48/45-FL and anti-6C-Mut antibodies, immunofluorescence assay (IFA) was performed with fixed and permeabilized mature gametocytes, gametes/zygotes (Fig. 3c). As expected from the SMFA results, anti-6C IgGs reacted to sexual-stage parasites, and negative control IgG did not. Corresponding to the ELISA results, anti-Pfs48/45-FL and anti-6C-Mut antibodies also recognized sexual-stage parasites judged by IFA. Taken together, it is suggested that anti-Pfs48/45-FL and anti-6C-Mut antibodies recognized epitope(s), which are likely to be non-functional, but presented both in the recombinant protein and parasites.

### Baculovirus expressed 6C fragment is homogeneous

Subsequent biochemical characterization focused on the 6C protein alone, given the successful induction of functional antibodies, while the 6C-Mut protein was utilized as a comparator. The yield of Pfs48/45-FL (~0.1 mg/L) limited its availability for use in further biochemical characterization presented here.

#### Intact mass spectrometry and peptide mapping analysis

**Figure 4:**
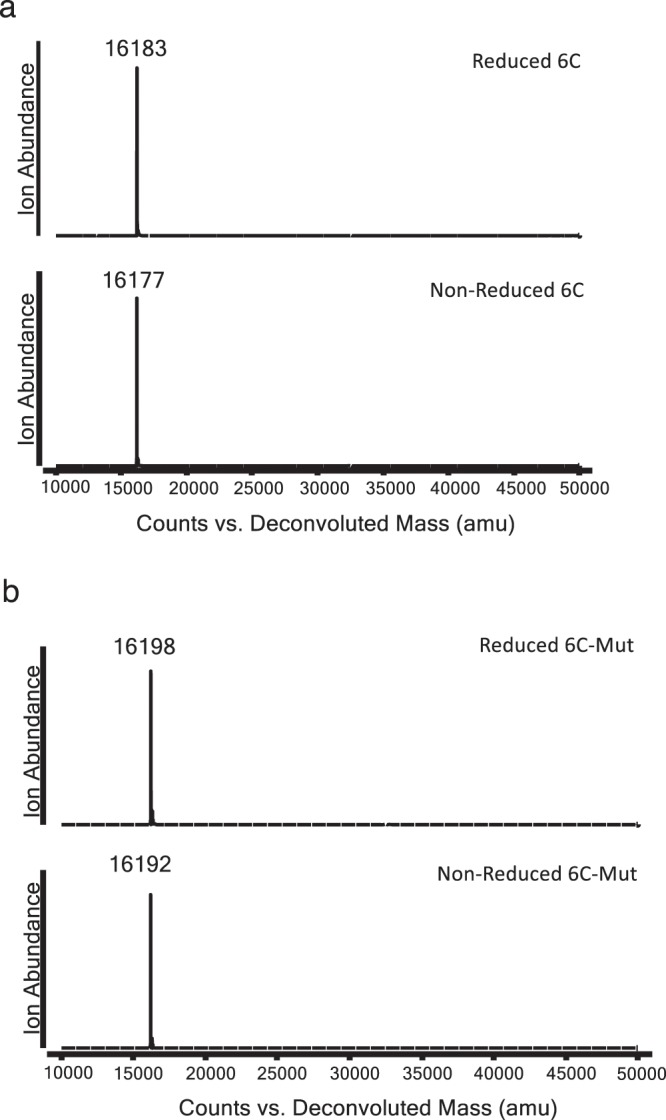
Representative intact mass analysis of Pfs48/45 6C and 6C-Mut proteins. Representative deconvoluted spectra (10–50 kDa) under reduced and non-reduced conditions. **(a)** Intact mass analysis of reduced & non-reduced 6C protein. Observed mass for reduced 6C was 16,183 ± 1 Da and non-reduced 6C was 16,177 ± 1 Da (n = 3) which corresponds to theoretical mass (assuming protein formed three intra-molecular disulfide bonds). **(b)** Intact mass analysis of reduced & non-reduced 6C-Mut protein. Observed mass for reduced 6C was 16,198 ± 1 Da and non-reduced 6C was 16,192 ± 1 Da (n = 3) which corresponds to theoretical mass (assuming protein formed three intra-molecular disulfide bonds).

To determine whether the baculovirus-expressed recombinant 6C was glycosylated, or altered through other post translational modification, intact mass spectrometry (MS) and LC-MS peptide mapping analyses were performed. The intact MS results (Fig. 4) indicated that the observed mass of the 6C fragment was +115 Da (Dalton) higher than its theoretical value of molecular mass. This mass difference was likely due to the inclusion of an N-terminal Asp residue from the last amino acid of the signal peptide, as reported previously^9,25^. After correcting for the Asp addition at the N-terminal, the predominant intact mass of 16,177 ± 1 Da (n = 3) matched the prediction for the non-reduced form of the 6C fragment. Upon reduction, an increase of six Daltons was observed, to 16,183 ± 1 Da (n = 3), matching the theoretical reduced form (Fig. 4a), and confirming the formation of three intra-molecular disulfide bonds. LC-MS peptide mapping (Supplementary Fig. S1) of the reduced and alkylated 6C fragment, utilizing GluC and Trypsin for proteolysis, confirmed 98% of the 6C sequences and no post-translational modifications were identified. These results combined demonstrated that tunicamycin effectively inhibited glycosylation of the non-mutated 6C molecule. Further, the intact mass of 6C-Mut (Fig. 4b) shows the oxidized and reduced form both matched the theoretical mass 16,192 ± 1 Da and 16,198 ± 1 Da (n = 3) with an Asp residue present at the N-terminus. In addition, these data demonstrate that the mutation of two sites effectively eliminated glycosylation of 6C-Mut.

#### Chromatography analysis of 6C and 6C-Mut proteins

**Figure 5:**
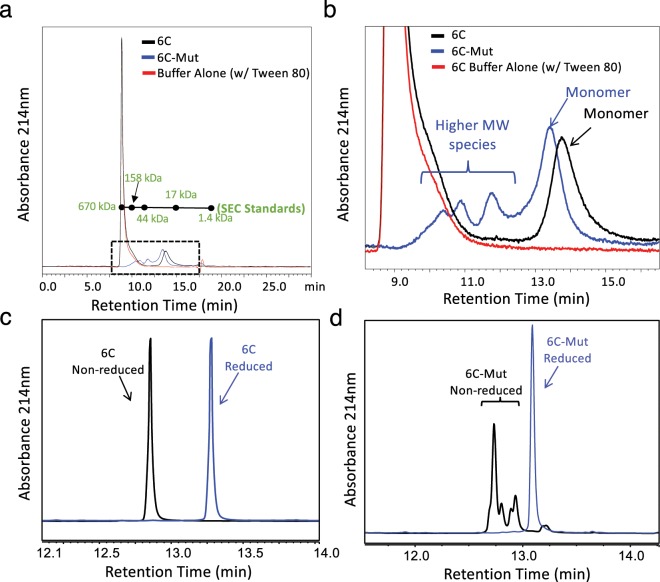
Representative chromatography analysis of Pfs48/45 6C and 6C-Mut proteins. (**a)** SE-HPLC analysis of 6C (black trace), 6C-Mut (blue trace) and buffer alone (red trace). Molecular weight retention times indicated according to Bio-Rad gel filtration standards. **(b)** Inset of SE-HPLC analysis with higher molecular weight and monomer peaks indicated. **(c)** RP-UHPLC of 6C (non-reduced and reduced). **(d)** RP-UHPLC of 6C-Mut (non-reduced and reduced).

Size exclusion (SE)-HPLC indicated that 6C was homogeneous and monomeric (Fig. 5a) eluting as a single peak at ~14 min. This retention time was consistent with a monomer based on the elution of gel filtration molecular weight (MW) standards. In contrast, 6C-Mut contained at least four species between ~670-17 kDa with only 59% monomeric protein (Fig. 5b). It should also be noted that substantial absorbance was observed within 8.5–11 min in the 6C sample, which was likely due to the high concentration of Tween 80 in the final formulation buffer.

In the reversed phase (RP)-HPLC analysis, the 6C protein was confirmed to be homogeneous with a single peak eluting under non-reducing or reducing conditions, at ~12.8 or ~13.3 min, respectively (Fig. 5c). Consistent with the SE-HPLC results, the 6C-Mut exhibited multiple species via RP-HPLC. Under non-reducing conditions, multiple peaks were observed for 6C-Mut (between 12.5–13.3 min) in the chromatogram. However, under reducing conditions, a single peak (~13.1 min) was observed. These results strongly suggest that the non-reduced form of the 6C-Mut contains multiple species with different disulfide linkages (Fig. 5d).

#### Isoelectric focusing

The biologically active 6C was evaluated for presence of isoforms. A single band was observed in the IEF gel for non-reduced 6C (Supplementary Fig. S2), which migrated between the 4.5–5.2 pI markers, a result consistent with the theoretical pI of 5.17. This result further confirmed that the 6C protein was present in a singular form.

### The 6C protein as expressed in baculovirus demonstrates predicted disulfide bond linkages

**Figure 6:**
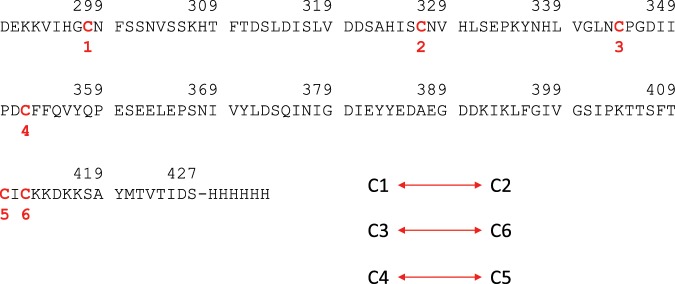
Disulfide pairing of Baculovirus expressed 6C as experimentally confirmed by LC/MS peptide mapping. Cys^298^, Cys^327^, Cys^344^, Cys^352^, Cys^410^ and Cys^412^ were denoted as C1 through C6. The majority of disulfide bonds in 6C (≥98%) are connected between C1 and C2, between C3 and C6 and between C4 and C5.

Given the importance of proper disulfide bond formation to ensure the native configuration of *Plasmodium* proteins and subsequent induction of functional antibodies, the disulfide linkages were investigated by two methods. First, to simply confirm the formation of disulfide bonds, the number of thiol groups exposed on the protein surface was measured. The analysis showed that 6C contains little or no free thiol (<5%) under both native-like and denaturing conditions (with and without 3 M guanidine-HCl), suggesting that all six cysteines were fully oxidized and likely paired by disulfides. Second, to discern the exact pairing of cysteines in the 6C protein, disulfide mapping was performed. The non-reduced and reduced 6C were subjected to an optimized thermolysin digestion method and the resulting peptides were analyzed by LC/MS peptide mapping. Table 1 shows the disulfide linked peptides that supported the pairing of the three disulfide bonds, primarily between Cys^298^ (C1) and Cys^327^ (C2), between Cys^344^ (C3) and Cys^412^ (C6) and between Cys^352^ (C4) and Cys^410^ (C5) along with the predicted theoretical peptide fragments (Table 2). A schematic representation of the amino acid sequence and elucidated disulfide mapping is provided in Fig. 6. This mapped pairing of cysteines for the baculovirus 6C matched the predicted disulfide bonding pattern of the cysteine-rich motif^13^.

**Table 1:** Disulfide peptides observed in the thermolysin digest of 6C.

RT Min	Expected monoisotopic mass Da	Identified peptide	Cys involved	Mass observed m/z 6C	Charge state	Calculated mass M	Delta mass ppm	Area
31.0	975.3903	I295-N299/I325-N328	C1 + C2 (Y3+Y12)	488.6922	2	975.3688	15.5	416
				976.3895	1	975.3817		
36.3	1074.4587	V294-N299/I325-N328	C1 + C2 (Y2-3+Y12)	538.2266	2	1074.4376	12.8	674
				1075.4601	1	1074.4523		
37.2	1157.4594	I295-N299/L342-D347	C1 + C3 (Y3+Y17)	579.7368	2	1157.4580	1.2	15
42.4	1256.5278	V294-N299/L342-D347	C1 + C3 (Y2-3+Y17)	629.2675	2	1256.5194	6.7	7
78.0	1246.5475	I295-N299/ I348-F353	C1 + C4 (Y3+Y18-20)	624.2809	2	1246.5462	1.1	2
86.5	1321.5683	L342-D347/I348-F353	C3 + C4 (Y17+Y18-20)	661.7900	2	1321.5644	3.0	4
28.1	1563.7749	L342-D347/I411-S418	C3 + C6 (Y17+Y38)	782.8868	2	1563.7580	11.8	1257
				522.2595	3	1563.7550		
79.5	926.3878	I348-C352/ F408-C410	C4 + C5 (Y18-19+Y37)	464.1954	2	926.3752	5.6	243
				927.3979	1	926.3901		
92.5	1073.4562	I348-F353/F408-C410	C4 + C5 (Y18-20+Y37)	537.7386	2	1073.4616	−4.3	951
				1074.468	1	1073.4601		
37.8	1505.7964	I348-C352/I411-S418	C4 + C6 (Y18-19+Y38)	753.9022	2	1505.7888	5.1	4
63.2	1652.8630	I348-F353/I411-S418	C4 + C6 (Y18-20+Y38)	827.4354	2	1652.8552	6.5	10
				551.9576	3	1652.8493		
18.2	1381.7058	I325-N328/I411-S418	C2 + C6 (Y12+Y38)	691.8593	2	1381.7030	−0.4	12
				461.5777	3	1381.7096		

Disulfide peptides and bonds observed for 6C in the thermolysin digest are presented. The delta mass error is the average of the errors for each of the charge state. Theoretical peptides of 6C are shown in Table 2.

**Table 2:** Theoretical fragments for thermolysin digest of 6C.

Fragment number	Amino acid	Theoretical mass	Amino acid
Y1	D+291-293	500.26	DEKK
Y2	294	117.08	V
Y3	295-299	542.23	IHGCN
Y4	300-303	453.19	FSSN
Y5	304-309	657.34	VSSKHT
Y6	310-313	468.19	FTDS
Y7	314-315	246.12	LD
Y8	316-317	218.13	IS
Y9	318	131.09	L
Y10	319-322	434.16	VDDS
Y11	323-324	226.11	AH
Y12	325-328	435.18	ISCN
Y13	329-330	254.14	VH
Y14	331-338	986.48	LSEPKYNH
Y15	339	131.09	L
Y16	340-341	174.1	VG
Y17	342-347	617.25	LNCPGD
Y18	348	131.09	I
Y19	349-352	446.18	IPDC
Y20	353	165.08	F
Y21	354-355	293.14	FQ
Y22	356-363	979.41	VYQPESEE
Y23	364-368	558.26	LEPSN
Y24	369	131.09	I
Y25	370-371	280.14	VY
Y26	372-375	461.21	LDSQ
Y27	376-377	245.14	IN
Y28	378-380	303.14	IGD
Y29	381-386	830.33	IEYYED
Y30	387-392	633.26	AEGDDK
Y31	393-394	259.19	IK
Y32	395	131.09	L
Y33	396-397	222.1	FG
Y34	398	131.09	I
Y35	399-401	261.13	VGS
Y36	402-407	645.37	IPKTTS
Y37	408-410	369.14	FTC
Y38	411-418	948.54	ICKKDKKS
Y39	419-420	252.11	AY
Y40	421-422	250.1	MT
Y41	423-424	218.13	VT
Y42	425-427+6H	1155.51	IDSHHHHHH

## Discussion

In these studies, we report for the first time the expression of Pfs48/45 proteins using the baculovirus system and their biological and biochemical evaluation. After expression, all three proteins were found in the cell pellet. This result suggests that in insect cells the inclusion of a signal peptide in the construct is not sufficient to direct secretion for the Pfs48/45 derived sequences. Further, no post-translational modifications were observed, indicating that *N*-glycosylation was successfully inhibited by the incorporation of tunicamycin during baculovirus expression. While produced in low yields, the Pfs48/45 full-length protein was expressed and reacted to conformational monoclonal antibodies, though it did not induce functional antibodies as determined by SMFA. Production of the 6C protein resulted in an un-optimized yield of 3 mg/L which allowed for detailed analysis of the biochemical properties.

We attempted to produce Pfs48/45 proteins in the non-glycosylated state presumably present in the parasite. In both the 6C and 6C-Mut proteins, *N*-glycosylation was successfully prevented by two independent approaches. The addition of 1 µg/mL tunicamycin resulted in undetectable glycosylation for the 6C protein as evaluated by intact mass spectrometric analysis and LC-MS peptide mapping. Further, the mutation of the two high probability glycosylation sites was also successful in preventing *N*-glycosylation for the 6C-Mut protein, as indicated by no detectable post translational modification based on intact mass. However, the mutation strategy did not produce a properly folded protein and multiple species were present as observed by SE and RP-HPLC. 6C-Mut was also not recognized by the conformational monoclonal antibody 85RF45.1, and it did not induce transmission reducing activity in mice.

Tunicamycin was previously used by Tonkin *et al*. to produce a secreted, soluble, non-glycosylated malaria antigen, Pf12, in the High Five cell/baculovirus expression system^30^. In those studies, the concentration of tunicamycin was 0.2 µg/mL and served as the basis for the concentrations tested here. They also reported in those studies that protein expression was severely attenuated with the addition of tunicamycin, consistent to our observations (Supplementary Table S1).Protein translocation through the ER, including its mechanisms and attenuation, while not fully known - have been proposed and described^31^. In the case of Pfs48/45 proteins produced here, even though the signal sequence was recognized by signal recognition particle and later cleaved, there was no guarantee of secretion. Similarly, Klaus *et al*. also faced an inefficient secretion of a recombinant vascular endothelial growth factor C (VEGF-C) to baculovirus/insect culture medium and was unable to improve secretion by altering culture conditions or signal peptides^32^. The lysate of the baculovirus infected insect cells, however, still constituted a valuable source of biologically active proteins^32^. Although the proteins reported here were successfully extracted from cell pellets using sarkosyl, a systematic and thorough evaluation of various extraction reagents and stabilization excipients were not explored, which merit further evaluation as part of future work.

In recent studies, Kundu *et al*. cloned the 6C fragment in HEK 293 cells without genetic modification of the *N*-glycosylation sites, N299 and N303, and the resulting crystal structure revealed that N303 is indeed glycosylated^16^. After EndoH treatment, the N-acetylglucosamine attached to N303 forms H-bonds with D390 and D391 of the 6C molecule. On the other hand, N299 was found not to be glycosylated with the side chain of N299 involved in H bonding with S301. These two *N*-glycosylation sites (N299 and N303) are not directly involved in the binding of 6C to 85RF45.1, but the results reported here, together with Kundu *et al*, suggest that amino acid sequence at these sites can play important roles in the proper folding of 6C^16^. While the glycosylation at these sites are not expected to occur in the parasite, our results also suggest that mutation of the primary sequence may also disturb higher order structure.

In this work, the 6C protein was the only protein that elicited transmission-reducing antibodies in mice. The 6C was also found to be properly folded and homogeneous in terms of charge, size and conformation. In contrast, the 6C-Mut was likely not in a native conformation, supported by a lack of 85RF45.1 binding and the presence of multiple chromatographic forms. Therefore, it is not surprising that no sera with transmission-reducing activity were identified for the 6C-Mut. It is not clear why the full-length version did not induce transmission reducing activity; perhaps potent epitope in the 6C domain was masked or out competed by other regions of the protein.

Our results on expression of full-length Pfs48/45 is consistent with the many previous attempts to generate this recombinant protein. While some of the Pfs48/45-FL protein was likely correctly folded in our analyses, additional strategies are needed to study this protein in greater detail before it may be considered as a viable vaccine candidate.

Given the presence of a pure, homogeneous protein for 6C fragment, coupled with the ability to elicit transmission reducing antibodies, we further confirmed the disulfide pairing of the recombinant protein. Our initial effort in mapping disulfides using GluC and trypsin digestion of non-reduced and reduced, alkylated 6C was not successful due to proximity of Cys^410^ and Cys^412^ (data not shown). Subsequently, improved methods were developed using thermolysin digestion which cleaves at the N-terminal of the hydrophobic amino acid residues^33^. Here, thermolysin cleaved multiple positions between Cys^344^ and Cys^412^, including Cys^410^ and Cys^412^ at the amino acid residue 411 (isoleucine). As shown in Table 1 and Fig. 6, Cys^410^(C5) and Cys^412^ (C6) are isolated to different peptides after thermolysis digestion, allowing us to accurately map the C3 and C6 connection and C4 and C5 connection unequivocally. These results coupled with the biochemical analysis (SE-HPLC, RP-HPLC, IEF) indicate the 6C protein is expressed in baculovirus as a homogeneous and properly folded molecule.

In this report, we demonstrated that baculovirus can express a properly folded 6C fragment without a fusion partner and tunicamycin was successful in producing a homogeneous, non-glycosylated 6C. The reported assessment of baculovirus-produced 6C represents a first step towards developing a TBV vaccine that targets the Pfs48/45 protein.

## Methods

### Baculovirus expression constructs

Three constructs of the Pfs48/45 protein were prepared for expression in baculovirus. “6C,” the C-terminal sequence of the gametocyte surface protein Pfs48/45 of 3D7 strain (ACCESSION Q816T1), containing six cysteines as part of a predicted cysteine-rich domain (aa 291–427); “6C-Mut,” which has two mutations of *N*-glycosylation sites, N299Q and N303D, incorporated in the 6C construct (aa 291–427); and “Pfs48/45-FL,” a full-length Pfs48/45 without the signal peptide and GPI anchor (aa 28–427).

Codon optimization for baculovirus expression was performed by DNA2.0 (now ATUM) with an additional N-terminal secretion signal (MKFLVNVALVFMVVYISYIYAD from Honeybee Melittin) and a C-terminal hexa-histidine tag. Genes were cloned into pFastBac donor vector (Invitrogen) with BamHI (5′) and EcoRI (3′) sites. The resulting plasmid pFastBac-48/45 constructs were sequence verified. Production of recombinant viruses followed the same procedure as described earlier^25^.

### Expression and purification of Pfs48/45 baculovirus proteins

Super Sf9 cells were seeded at 1 × 10^6^ cells/mL in ESF 921 medium (Expression Systems, CA). MOI of one was used to infect a 10 L super Sf9 wave culture and tunicamycin was added at a final concentration of 1 µg/mL. At 96 h post infection, culture was harvested, and the cell pellet was resuspended in lysis buffer (20 mM Tris-HCl, 2% sarkosyl, protease inhibitor tablets, pH 8.0) and processed with a high-pressure homogenizer (Panda plus). The soluble fraction was recovered by centrifugation, diluted with 20 mM Tris-HCl, 300 mM NaCl, 5 mM imidazole, pH 8.0 (buffer A) and clarified with 0.22 μm filtration.

The clarified supernatant was loaded on a 20 mL Ni-NTA column (1.6 × 10 cm, His-60, Clontech). The wash steps were performed with ten column volume (CV) of Buffer A with 20 mM imidazole and 0.2% Tween-80. The protein was eluted with a 20 to 100% gradient of buffer B (20 mM Tris-HCl, 500 mM NaCl, 500 mM imidazole, 0.2% Tween-80, pH 8.0). Pooled eluents from Ni-NTA column were loaded onto a Superdex 75 column (GE Healthcare, 2.6 × 60 cm, 300 mL) and buffer exchanged into 20 mM HEPES, 150 mM NaCl containing 0.2% Tween-80 (pH 7.5). For the Pfs48/45-FL protein, a Superdex 200 was used after His-60 column. The eluents were collected, analyzed with SDS-PAGE, and final pool selected based on purity.

### SDS-PAGE and Western blotting (anti-His)

SDS-PAGE and Western blot with Penta-His antibody (Qiagen) were performed as described earleir^9^.

### Western blotting (mAb 85RF45.1)

Following SDS-PAGE, proteins were transferred onto PVDF membrane and blocked in 1% skim milk in Tris buffered saline containing 0.05% Tween-20 (TBST) at room temperature for one hour. Primary antibody at a 1:1,000 dilution of mAb 85RF45.1 (1 mg/mL) in TBST was added and incubated for 1 h at room temperature. Membranes were washed with TBST (3X for 10 min) and secondary antibody, 1:5,000 dilution of goat anti-rat IgG-alkaline phosphatase (Bio-Rad) in TBST was incubated at room temperature for 1 h. Membranes were washed with TBST (3X for 10 min), developed using alkaline phosphatase substrate kit (Bio-Rad), followed by a water wash to stop the reaction and air-dried.

### Protein concentration determination

Recombinant Pfs48/45 proteins contain no tryptophan residue and UV at A_280_ was not ideal for protein determination. The BCA assay kit (Thermo) was therefore used according to manufacturer’s instructions.

### Mouse immunization

All animal experiments were performed in accordance with the animal study protocol (LMVR 10E) which was reviewed and approved by the NIAID Animal Care and Use Committee. CD-1 mice (n = 10 per group) were immunized intramuscularly with Pfs48/45-FL (3 μg per dose), 6C-Mut (10 μg) or 6C (3 or 10 μg) recombinant protein formulated with Montanide ISA720 (SEPPIC Inc., Fairfield, NJ) on days 0 and 21. Serum samples were collected on day 42. As a negative control, a group of mice (n = 10) were immunized with ISA720 alone on the same schedule.

### IgG purification, SMFA and ELISA

The biological activity of anti-Pfs48/45 IgGs was tested by SMFA at 750 μg/mL with complement in the first assay, and the two positive IgGs (anti-6C IgGs) from the first assay were further evaluated at 750, 250 and 83 μg/mL with complement in a second assay. In each assay, the anti-adjuvant IgG was tested at 750 μg/mL as a negative control. The standardized methodology for performing the SMFA has been described previously^34^. In brief, 16–18-day old gametocyte cultures of the *P. falciparum* NF54 line were mixed with test IgGs at indicated concentrations, and fed to *Anopheles stephensi* mosquitoes. All feeding experiments were performed with human complement and n = 20 mosquitoes per group were examined 8 days after feeding experiment for oocyst count.

Basic methodology of regular ELISA has been described elsewhere^35^. For Pfs48/45-FL and 6C-Mut groups, individual antisera were tested against both the corresponding immunogen and 6C protein. For 6C (3 µg) and 6C (10 µg) groups, ELISA units were determined against the 6C protein alone. The antisera from ISA720 control group were tested against all three proteins.

### Statistics

Percent inhibition in mean oocyst intensity (%TRA) of a test sample was calculated against a control sample examined in the same feeding experiment. The best estimate and 95% confidence intervals (95%CIs) of %TRA, and p-value (whether the inhibition is significantly different from no inhibition) for each test condition was calculated using a negative binomial model with zero inflation model, as described previously^36^. All statistical analysis was performed in R (version 3.4.1) or Prism 7 (GraphPad Software), and p-values < 0.05 were considered significant.

### Immunofluorescence assay (IFA)

The IFA was performed as described previously^9^ with minor modifications. In this study, mature gametocytes were incubated with 100% human serum at 19 °C for 20 minutes to induce gametes and zygotes, and IFA slides prepared using the mixture of gametocytes, gametes and zygotes. The cells were fixed and permeabilized with a 1:1 mixture of methanol-acetone, and the slides were blocked by PBS with 3% skim milk. The primary antibodies (purified IgG from each group, which were used for SMFA) were incubated at 1 µg/mL for 1 hour at 37 °C, then stained with secondary antibody and DAPI (4′,6-diamidino-2-phenylindole) as described previously. All images were captured on a Lecia SP8 confocal microscope with LAS X software version 3.5.5.19976. Images were deconvolved using Huygens essential software version 19.04.0p2 64b.

### Intact mass spectrometry

The 6C or 6C-Mut protein was incubated in either water (non-reduced) or 50 mM DTT (reduced) for 30 min at 60 °C and desalted on a C4 column (Waters Corp.) prior to MS analysis. The intact mass of the protein was measured using a Model 6230B time-of-flight (ToF) mass spectrometer (Agilent Technologies). MS^1^ spectra were acquired over a mass range of 400–3200 m/z, with a scan rate of one spectra/sec. Protein deconvolution was performed using MassHunter (Agilent Technologies).

### LC-MS peptide mapping

Recombinant 6C was bound to a strong anion exchange spin filter (Pierce), washed with phosphate and eluted with 1 M NaCl. The sample was reduced with 5 mM DTT for 30 min at 60 °C and alkylated with 10 mM iodoacetamide at 37 °C for 30 min in the dark. The reduced and alkylated 6C was digested with 1:1 mixture of GluC and Trypsin at 37 °C overnight and subjected to LC/MS for peptide identification using an Ultimate 3000 UHPLC system (Thermo Scientific) with a 1.7 µm C18 column (2.1 × 150 mm, Thermo) at a flow rate of 0.2 mL/min. C18 column and autosampler temperatures were set at 50 °C and 5 °C, respectively. Mobile phase (A) consisted of Water with 0.04% trifluoroacetic acid (TFA) and mobile phase (B) consisted of Acetonitrile with 0.04% TFA. MS range of 400–1900 m/z was used. Peptides were identified using MS^1^ and MS^2^ data and PepFinder (Thermo Scientific).

### SE-HPLC

SE-HPLC used the same method as described earlier^9^ with the exception of the mobile phase, which was 20 mM HEPES, 150 mM NaCl, pH 7.5.

### RP-HPLC

Proteins were incubated with either 10% water (non-reduced) or 100 mM DTT (reduced) for 30 min at 60 °C, centrifuged for 5 min at 14,000 × g, and then subjected to RP-UHPLC using the same method as described earlier^25^. Triplicate experiments were performed.

### Isoelectric focusing

Precast CleanGel IEF with Pharmalyte 4–6.5 (GE Healthcare) was used with 5 µg of non-reduced 6C sample and electrophoresis conducted at 2000 V, 7 mA, 7 W for 30 min. The gel was fixed with 20% TFA for 30 min, stained with Coomassie R250 for 30 min, and then detained with 40% methanol and 10% acetic acid.

### Free thiol determination

Free thiol (number of free cysteine residues) was measured using Ellman’s reagent (Thermo Scientific) following manufacturer’s instructions. The 6C protein was concentrated to 1.4 mg/mL (~85 µM) prior to thiol assay. A standard curve was constructed using known concentrations of reduced glutathione. Absorbance was measured at 412 nm.

### Disulfide mapping

The 6C protein was buffer exchanged into 50 mM Tris buffer containing 10 mM CaCl_2_, pH 7 without denaturation or alkylation. Samples were digested with thermolysin (Promega) at a 1:10 enzyme to protein ratio overnight at 37 °C. A portion of the digest was reduced using 20 mM TECP [Tris (2-carboethyl) phosphine] at 37 °C for 30 min. The digested peptides were then subjected to LC/MS using a C18 column (2.1 × 150 mm, 1.7 µm, Waters BEH) set to 40°C. Mobile phases were A (water + 0.05% TFA) and B (acetonitrile + 0.05% TFA) and the peptides were eluted using a 1–20% B gradient over 90 min at 0.2 mL/min flowrate. Differences between the reduced and non-reduced samples were used to locate the disulfide peptides. Peptides were identified using a Waters Synapt G2 or QToF Premier mass spectrometer in positive-ion mode and a mass range of 100–3000 Da. The identities of the disulfide peptides observed in the thermolysin digest were confirmed with MS^e^ data. Peptides were considered confirmed if at least two a, b or y ions were observed for each individual peptide in the disulfide peptide pair. A control protein, R0.6C^20^ was used to develop and optimize the mapping method.

## Supplementary information

Supplementary Material.

## Data Availability

The datasets generated during and/or analysed during the current study are available from the corresponding author on reasonable request.
