# Supplementary material for: A C-terminal Pfs48/45 malaria transmission-blocking vaccine candidate produced in the baculovirus expression system
Source: Sci Rep. 2020 Jan 15;10:395. doi: 10.1038/s41598-019-57384-w (PMC6962329; doi:10.1038/s41598-019-57384-w)
Supplement: Supplementary file 1 — Supplementary Material. [file 41598_2019_57384_MOESM1_ESM.docx]

**Supplementary Material**

**A C-terminal Pfs48/45 malaria transmission-blocking vaccine candidate produced in the baculovirus expression system**

Shwu-Maan Lee^1^*, John M. Hickey^2^, Kazutoyo Miura^3^, Sangeeta B. Joshi^2^, David B. Volkin^2^, C. Richter King,^1^ Jordan L. Plieskatt^1^

|  | **Millions of Super Sf9 cells** | | |
| --- | --- | --- | --- |
| **Hours Incubation** | **Control (No tunicamycin)** | **0.2 µg/mL Tunicamycin** | **1 µg/mL Tunicamycin** |
| 48 | 3.5 | 2.2 | 1.3 |
| 72 | 6.5 | 4.8 | 0.8 |

**Supplementary Table S1.** Effect of Tunicamycin on cell growth of super Sf9 cells.

| **Name** | | **Concentration (µg /mL)** | **Mean oocyst** | **%TRA** | **(95%CI)** | **p-value** |
| --- | --- | --- | --- | --- | --- | --- |
| ***Feed #1*** | | | | | | |
|  | Adjuvant alone | 750.0 | 27.9 | - | - | - |
|  | Pfs48/45-FL (3 µg) | 750.0 | 29.7 | -6.6 | (-133 to 51) | 0.873 |
|  | 6C-Mut (10 µg) | 750.0 | 25.9 | 7.2 | (-117 to 58) | 0.881 |
|  | 6C (3 µg) | 750.0 | 0.5 | 98.4 | (95 to 100) | 0.001 |
|  | 6C (10 µg) | 750.0 | 0.5 | 98.2 | (98 to 99) | 0.001 |
| ***Feed #2*** | | | | | | |
|  | Adjuvant alone | 750.0 | 7.1 | - | - | - |
|  | 6C (3 µg) | 750.0 | 0.8 | 89.4 | (76 to 96) | 0.001 |
|  |  | 250.0 | 4.1 | 43.0 | (-35 to 76) | 0.184 |
|  |  | 83.3 | 5.3 | 25.4 | (-87 to 71) | 0.530 |
|  | 6C (10 µg) | 750.0 | 0.6 | 91.5 | (80 to 97) | 0.001 |
|  |  | 250.0 | 5.0 | 29.6 | (-65 to 71) | 0.416 |
|  |  | 83.3 | 6.3 | 11.3 | (-110 to 60) | 0.775 |

**Supplementary Table S2.** SMFA results with IgGs from mice immunized with Pfs48/45-FL, 6C-Mut and 6C.

**Supplementary Figure. S1: LC-MS peptide mapping of Baculovirus expressed 6C showing 98% sequence confirmation.** (a) GluC and trypsin (1:1) digested peptides from reduced alkylated 6C were separated on a C18 RP HPLC column. (b) The primary sequence of 6C showing 98% sequence coverage from peptide mapping analysis of the reduced form of the protein.


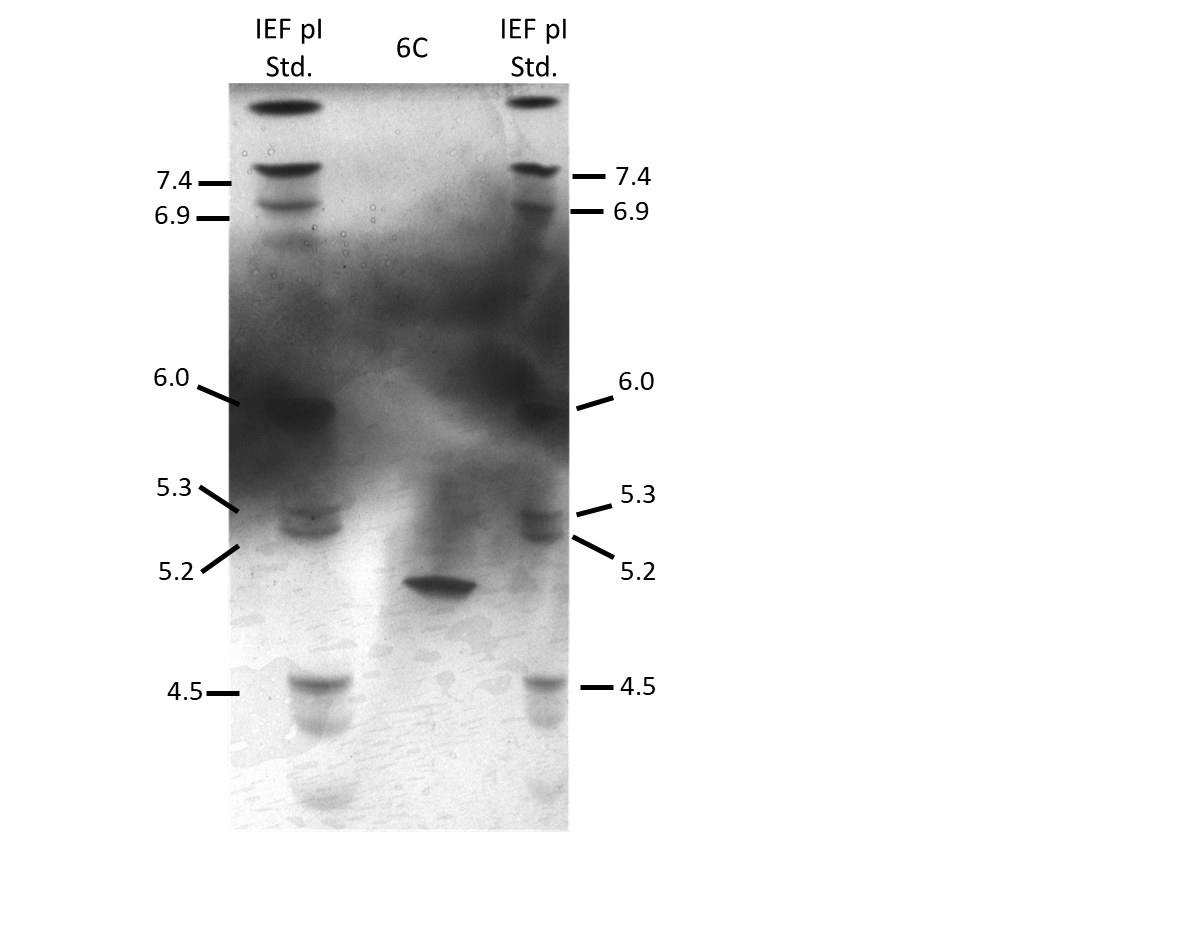


**Supplementary Figure S2. Isoelectric focusing gel analysis of 6C.** The theoretical pI of 6C is 5.2. One major band is observed for non-reduced 6C migrating between the 4.5 and 5.2 pI markers. Image was cropped at time of acquisition to lanes of interest. Image has not been manipulated in any further manner.
